# Awareness of the Adult Population Toward Colorectal Cancer in Qassim Region, Saudi Arabia

**DOI:** 10.7759/cureus.33477

**Published:** 2023-01-07

**Authors:** Sultan Alsaigh, Ftoun A Almuhaimeed, Najla A Alsaqabi, Alhanouf M Alwehaibi, Hakem S Al-Mutairi

**Affiliations:** 1 General Surgery, King Fahad Specialist Hospital, Buraidah, SAU; 2 General Surgery, Qassim University, Qassim, SAU; 3 General Surgery, Ministry of Health Holdings, Qassim, SAU

**Keywords:** cancer, colonoscopy, adult population, awareness, colon, colorectal cancer

## Abstract

Introduction

Colon and rectal cancer are usually grouped together as they share similar features. They are the third most common cancer in the world and the second most common cancer in the Kingdom of Saudi Arabia (KSA). Awareness and preventive screening programs play a vital role in early diagnosis and improving the survival rate of such patients. This study aimed to assess the awareness and knowledge of colorectal cancer (CRC) among the adult population in the Qassim region of Saudi Arabia.

Methods

This is a cross-sectional study conducted among the adult population living in the Qassim region of Saudi Arabia. A self-administered questionnaire was disseminated among the adult population using WhatsApp and Twitter. The questionnaire contained demographic data and questions to assess the awareness, knowledge, and attitude regarding CRC risk factors and complications and the importance of screening.

Results

A total of 431 respondents were involved. The most common symptoms of colon cancer were abdominal pain and change in bowel habits. Risk factors associated with colon cancer were inflammatory bowel disease and fatty food. Colonoscopy was the most dominant choice for the detection of colon cancer.

Conclusion

The awareness of the adult population toward CRC was deficient. Better awareness can be predicted among individuals with better education. Addressing the gaps in awareness is vital to alleviate fears and misconceptions surrounding this disease.

## Introduction

Colorectal cancer (CRC) is characterized by abnormal cancerous division of cells in the colon or rectum [1]. CRC usually initiates as a polyp, which is a non-cancerous growth in the inner mucosal lining of the colon or rectum [2]. Around 10% of non-cancerous polyps progressed to invasive cancer into the wall of the colon or rectum, thereby invading the nearby lymph nodes and lymph vessels. In later stages, it can spread to other organs, such as the liver, lungs, and peritoneum [3]. There are several risk factors associated with CRC, including low exercise in daily routine, high amount of red meat, low intake of vegetable and fruit consumption in daily intake, and family history of inflammatory bowel disease and CRC [4]. Early diagnosis and screening of CRC are reported to be associated with increased survival and reduced mortality [5]. Levels of awareness and knowledge about CRC have been found to vary globally. A study revealed a high level of knowledge about risk factors and screening of CRC in the United States population [6]. In contrast, a study among the Malaysian population exhibited dangerously alarming levels of unawareness with around 38% of study participants having zero knowledge about warning signs and risk factors associated with CRC [1]. In Saudi Arabia, CRC is the second most occurring cancer but the survival rate of CRC is reportedly low as compared to other countries, i.e., 44% survival rate after five years as compared to a 60% survival rate of CRC in the United States [7]. One of the leading factors associated with a low survival rate among the Saudi population is a low level of awareness and participation in preventing screening [8].

Colon and rectal cancer are usually grouped together as they share similar features. They are the third most common cancer in the world and the second most common cancer in the Kingdom of Saudi Arabia (KSA). Awareness and preventive screening programs play a vital role in early diagnosis and improving the survival rate of such patients. This study aimed to assess the awareness and knowledge of CRC among the adult population in the Qassim region of Saudi Arabia.

## Materials and methods

This was a cross-sectional study carried out among the adult population in the Qassim region of Saudi Arabia, which has almost 1.5 million people living in it. We included all adults regardless of age or gender who live in the Qassim region and were willing to fill out the questionnaire to assess the awareness, knowledge, and attitude regarding CRC risk factors and complications and the importance of screening. Non-Saudis or people living in another region were excluded. The sample size after using the total enumeration method was 600 participants with a 95% confidence interval and a margin of error of 4%. Electronic questionnaires were distributed through social media platforms.

Data collection tools and instruments

We used a validated questionnaire that was used in a previously published study [9] with some modifications. A certified translator translated the questionnaire from English to Arabic, the public language. The questionnaire was converted to an online version by using Google Forms (Google, Mountain View, CA). The questionnaire contains demographic data and quotations to assess the awareness, knowledge, and attitude regarding CRC risk factors and complications and the importance of screening.

Ethical considerations

We obtained ethical approval from the Qassim Research Ethics Committee (IRB no.: 607/44/5837). The data were used specifically for research purposes, and all sensitive information was kept confidential.

Data analysis

We used SPSS (IBM Corp., Armonk, NY) for statistical analysis. Percentages and frequencies were used to describe categorical data such as gender, educational level, and age. Mean and standard deviations were used to describe numerical data. For the two open-ended response questions, we sorted them by similarity and picked the most frequent answers. We considered all data with a p-value of less than 0.05 statistically significant.

## Results

A total of 431 respondents were involved in the study. The most common symptoms of colon cancer were abdominal pain (71.5%) and change in bowel habits (61%). Risk factors associated with colon cancer were inflammatory bowel disease (56.8%) and fatty food (50.1%). Colonoscopy was the most dominant choice for the detection of colon cancer (83.1%). The overall mean awareness score was 7.28 (SD: 2.39), and poor knowledge levels were shown among 57.3%; 38.5% were moderate and only 4.2% had good awareness levels. Being more educated was the factor associated with an increased awareness score.

A total of 431 participants responded to our survey. Table 1 presents the basic demographic characteristics of the participants. Approximately 60.1% of participants were single and more than three-quarters (77%) of participants had bachelor’s degrees. In addition, 60.6% indicated that the incidence of color cancer was widely prevalent.

**Table 1:** Participants’ socio-demographic characteristics (n = 431) PhD: Doctor of Philosophy.

Study variables	N (%)
Marital status	
Single	259 (60.1%)
Married	172 (39.9%)
Educational level	
Secondary	75 (17.4%)
Bachelor’s degree	332 (77.0%)
Master’s or PhD	24 (05.6%)
The incidence of colon cancer	
Widely	261 (60.6%)
Average	155 (36.0%)
Rare	15 (03.5%)

**Figure 1:**
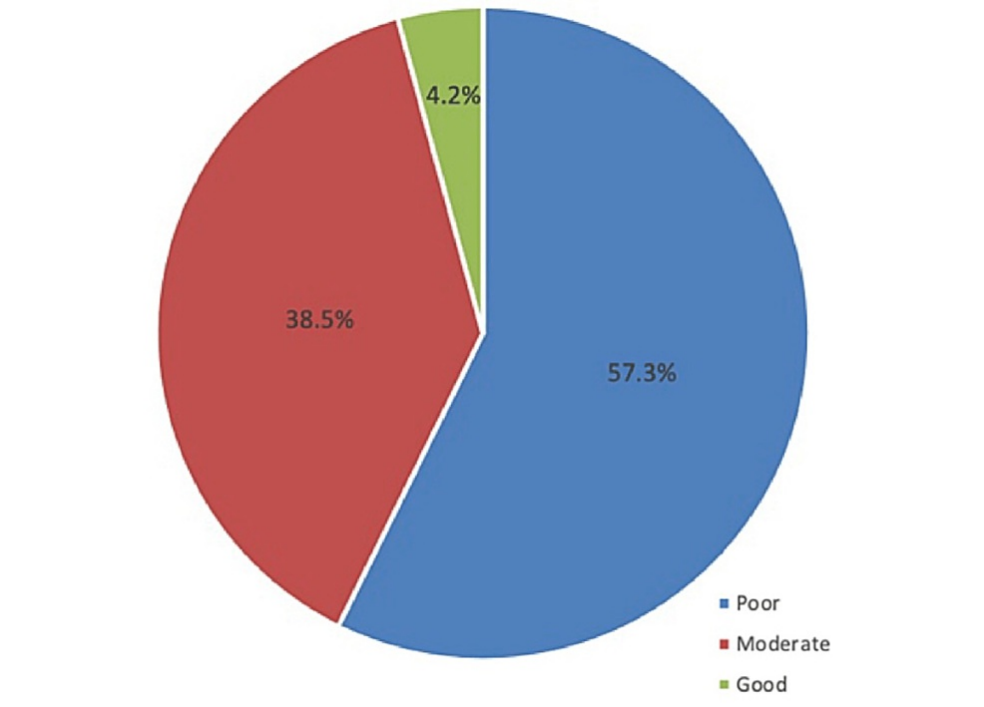
Level of awareness toward colorectal cancer

The assessment of awareness of CRC is presented in Table 2 and Figure 1. It was observed that respondents who knew the definition of the colon were 43.4%. Of the respondents, 38.1% knew that the most common function of the colon was water reabsorption. Only 25.3% were aware of the correct age for screening CRC tests. The most common symptom of colon cancer was abdominal pain (71.5%), followed by a change in bowel habits (61%) and the presence of blood in the stool (52%). The most common risk factor of colon cancer was inflammatory bowel disease (56.8%), followed by fatty food (50.1%), and a family history of colon cancer (46.2%). Respondents were confident that the most common method to detect colon cancer was colonoscopy (83.1%). Nearly 60% knew that colon cancer can be cured if detected early and 38.1% believed that there was a relationship between colon cancer and irritable bowel syndrome. The overall mean awareness score was 7.28 (SD: 2.39) with poor, moderate, and good awareness levels detected among 57.3%, 38.5%, and 4.2%, respectively.

**Table 2:** Assessment of awareness toward colorectal cancer (n = 431) ^†^ Variable with multiple response answers. * Indicates correct answer.

Awareness questions	N (%)
Definition of colon	
Large intestine*	187 (43.4%)
Small intestine	53 (12.3%)
The last part of the large intestine	119 (27.6%)
The last part of the small intestine	72 (16.7%)
Function of colon	
Digestion of food	156 (36.2%)
Waste storage	55 (12.8%)
Water reabsorption*	164 (38.1%)
Does not have any function	56 (13.0%)
When do you screen for colorectal cancer?	
At the onset of symptoms	273 (63.3%)
At the age of 20 years	43 (10.0%)
At the age of 50 years*	109 (25.3%)
At the age of 70 years	06 (01.4%)
Symptoms of colon cancer^†^	
Abdominal pain*	308 (71.5%)
Change in the bowel habit*	263 (61.0%)
Presence of blood in stool*	224 (52.0%)
Significant weight loss over a certain period of time*	203 (47.1%)
Nausea and vomiting*	152 (35.3%)
Yellow discoloration of the eyes and skin	55 (12.8%)
Itchy skin	21 (04.9%)
Risk factors for colon cancer^†^	
Inflammatory bowel disease*	245 (56.8%)
Fatty food*	216 (50.1%)
Family history of colon cancer*	199 (46.2%)
Smoking*	181 (42.0%)
Colon polyps*	95 (22.0%)
Used for early detection of colon cancer^†^	
Fecal occult blood test (FOB)*	168 (39.0%)
Colonoscopy*	358 (83.1%)
X-ray	124 (28.8%)
Can colon cancer be cured when it is detected?	
Yes*	258 (59.9%)
No	18 (04.2%)
I don’t know	155 (36.0%)
Do you think there is a relationship between colon cancer and irritable bowel syndrome?	
Yes*	164 (38.1%)
No	116 (26.9%)
I don’t know	151 (35.0%)
Total awareness score (mean ± SD)	7.28 ± 2.39
Level of awareness	
Poor	247 (57.3%)
Moderate	166 (38.5%)
Good	18 (04.2%)

As shown in Table 3, a higher mean awareness score was associated with being more educated (Z = 3.049; p = 0.002) while the difference in the awareness score of single and married was not statistically significant (p = 0.099).

**Table 3:** Difference in the score of knowledge according to marital status and education (n = 431) ^§^ P-value has been calculated using Mann-Whitney Z-test. ** Significant at p < 0.05 level.

Factor	Awareness score (17), mean ± SD	Z-test	P-value^§^
Marital status			
Single	7.43 ± 2.46	1.651	0.099
Married	7.06 ± 2.26		
Educational level			
Secondary	6.49 ± 2.09	3.049	0.002**
Bachelor's degree or higher	7.45 ± 2.41		

## Discussion

This study attempted to evaluate the awareness of CRC among the adult population living in the Qassim region of Saudi Arabia. The awareness of the study population regarding CRC was inadequate. Approximately 57.3% were classified as having poor awareness, 38.5% had moderate awareness, and only 4.2% were considered as having a good awareness level (mean score: 7.28 and SD: 2.39, out of 17 points). Consistent with these findings, several papers documented a poor level of awareness of CRC, including its symptoms, risk factors, and screening tests [10-12]. However, a study conducted among 1,070 participants in Riyadh [13] found that the knowledge of the population regarding CRC was fair. They further added that although older individuals and those with higher education tended to answer questions correctly more often, there were some misconceptions regarding universally accepted screening protocols, symptoms, and a general understanding of CRC among the population living in Riyadh, Saudi Arabia. The lack of understanding about the disease is a concern that can be addressed through educational campaigns targeting people with the most limited knowledge and those with a lack of access to medical care.

A survey conducted among the general population in the Western region of Saudi Arabia [8] discovered that educational level played an influential role in the level of awareness. This has been concurred by a previous study [12], where participants with a higher education level were found to have a higher level of knowledge regarding the warning signs of CRC. Our study led to the same scenario, as we found out that respondents with higher educational degrees demonstrated better awareness scores compared to less-educated respondents; however, unlike a study [14] where the authors observed a significant relationship between marital status and the knowledge toward CRC, in our study, we found no significant association between marital status and awareness score. Therefore, more investigations are warranted to determine the true effect of marital status on the awareness level of the sample population.

Based on the understanding of our population, abdominal pain (71.5%), change in bowel movement (61%), and blood in the stool (52%) were the most common symptoms of colon cancer. However, their knowledge about weight loss (47.1%) and nausea and vomiting (35.3%) as symptoms of CRC was suboptimal, which needed education to narrow the gaps. Consistent with our findings, abdominal pain was also observed as the most common symptom among the urban population living in the Klang Valley, Malaysia [14]. Similarly, unexplained weight loss, blood in the stool, and lumps in the abdomen were the most commonly cited symptoms of CRC [11]. However, in studies done in Ethiopia [15] and Saudi Arabia [16], the most common symptom of CRC was blood in the stool, followed by abdominal pain and changes in bowel habits.

Regarding the risk factors for CRC, based on the knowledge of our population, inflammatory bowel disease (56.8%), fatty food (50.1%), and family history of colon cancer (50.1%) were identified as the most common risk factors for the disease. There were conflicting reports among publications about the risk factors of CRC. For example, in a study done in Qatar [12], daily eating of processed meat, use of tobacco, and drinking alcohol were the most commonly known risk factors for CRC, while in a paper published in Malaysia [14], having other bowel diseases, low fiber diet, and obesity were determined as the risk factors for CRC. Other studies indicated family history, age of more than 50 years, and smoking as the most commonly known risk factors for the disease [10,14-16]. It is important to establish the most common risk factors for CRC since this could guide the persons who are at high risk, which may lead to an increase in their willingness to undergo a screening test.

A previous study [13] indicated that the general population living in Riyadh, KSA knew that early detection of CRC through colonoscopy was significantly associated with higher survival rates. In our study, colonoscopy was also the dominant choice for the detection of CRC; however, in another study done among older Saudis [10], endoscopy was the most common screening method (72.4%) followed by an occult fecal blood test (61.8%). In Lebanon, 50% of patients who were planning to get screened in the future selected the fecal occult blood test stating that being easier was the most prominent reason for preference while for those who selected colonoscopy (42%), accuracy was the main reason for a preference [11]. Determining the reason behind the preferential screening method illuminates the factors that have the highest influence on screening options and therefore may guide future endeavors to increase screening rates among the Saudi adult population.

There were several determinants of awareness where our population demonstrated poor knowledge. For instance, only 43.4% of the subjects knew about the correct definition of colon and they showed a lack of knowledge about its correct function. Furthermore, only 25.3% believed that regular screening for CRC should start at the age of 50 years and only 38.1% believed that there is a relationship between colon cancer and irritable bowel movement. This is almost comparable to another study where according to their reports, only 51.7% were aware that the colon was the large intestine; however, most respondents believed that CRC is preventable and 97.4% thought that CRC could be cured if detected early. In our study, however, only 59.9% believed that colon cancer can be cured, which was lower than the previous report [13].

Limitations

Respondent biases in the surveys are possible. So generalization of this study's results should be done carefully.

## Conclusions

The awareness of the adult population toward CRC was deficient. Better awareness can be predicted among individuals with better education, but marital status did not influence the level of awareness. It is necessary to address the lack of awareness among the general population. Health education and awareness campaigns are the most important methods to bridge the gap in awareness. Finally, the role of healthcare providers in promoting awareness is vital specifically among individuals with the most limited knowledge about the disease.
